# A simple nomogram score for screening patients with type 2 diabetes to detect those with hypertension: A cross-sectional study based on a large community survey in China

**DOI:** 10.1371/journal.pone.0236957

**Published:** 2020-08-07

**Authors:** Mingyue Xue, Li Liu, Shuxia Wang, Yinxia Su, Kun Lv, Mingchen Zhang, Hua Yao

**Affiliations:** 1 College of Public Health, Xinjiang Medical University, Urumqi, China; 2 Hospital of Traditional Chinese Medicine Affiliated to the Fourth Clinical Medical College of Xinjiang Medical University, Urumqi, China; 3 Center of Health Management, The First Affiliated Hospital, Xinjiang Medical University, Urumqi, China; 4 The First Affiliated Hospital of Xinjiang Medical University, Urumqi, China; University of Zurich, SWITZERLAND

## Abstract

**Objectives:**

Compared with unaffected individuals, patients with type 2 diabetes (T2DM) have higher risk of hypertension, and diabetes combined with hypertension can lead to server cardiovascular disease. Therefore, the purpose of this study was to establish a simple nomogram model to identify the determinants of hypertension in patients with T2DM and to quickly calculate the probability of hypertension in individuals with T2DM.

**Materials and methods:**

A total of 643,439 subjects participating in the national physical examination has been recruited in this cross-sectional study. After excluding unqualified subjects, 30,507 adults with T2DM were included in the final analysis. 21,355 and 9,152 subjects were randomly assigned to the model developing group and validation group, respectively, with a ratio of 7:3. The potential risk factors used in this study to assess hypertension in patients with T2DM included questionnaire investigation and physical measurement variables. We used the least absolute shrinkage and selection operator models to optimize feature selection, and the multivariable logistic regression analysis was for predicting model. Discrimination and calibration were assessed using the receiver operating curve (ROC) and calibration curve.

**Results:**

The results showed that the major determinants of hypertension in patients with T2DM were age, gender, drinking, exercise, smoking, obesity and atherosclerotic vascular disease. The area under ROC curve of developing group and validation group are both 0.814, indicating that the prediction model owns high disease recognition ability. The *p* values of the two calibration curves are 0.625 and 0.445, suggesting that the nomogram gives good calibration.

**Conclusion:**

The individualized nomogram model can facilitate improved screening and early identification of patients with hypertension in T2DM. This procedure will be useful in developing regions with high epidemiological risk and poor socioeconomic status just like Urumqi, in Northern China.

## Introduction

Diabetes, cancer and cerebrovascular and cardiovascular diseases (CCVd) are known as the major chronic diseases in the world, which threatening human life and people's physical and mental health with the rising trend. Blood glucose and blood pressure control are the priority of national public health in China [1]. The seventh statistical result of the International Diabetes Federation (IDF) shows that there are 425 million people suffering from diabetes worldwide [2]. The prevalence of hypertension should be paid more attention as well, a nationwide survey shows that 29.6%(about 311.9 million) of Chinese adults over 18 years old have high blood pressure, and 41.3%(about 244.5 million) are in pre-hypertension (pre-HTN) [3], while the treatment and control rate in all hypertensives are less than 30% and 10%, respectively [4].

In general, diabetes and hypertension coexist, and the two diseases will aggravate each other [5, 6]. A national survey of outpatients and community residents in China shows that nearly a quarter of diabetic patients have hypertension at the same time, but the control rate of blood pressure is low [4, 7, 8]. As we know, diabetes and hypertension are the main risk factors of cardiovascular disease, and their combination can lead to severer atherosclerosis. Therefore, the early detection and economical screening for hypertension in diabetic patients will be a more urgent challenge for doctors all over the world.

At present, many studies aim at the situation that the prevalence of other diseases increases while both hypertension and diabetes show up, such as cardiovascular disease [9], kidney disease [10], heart disease [11], etc. There are also studies exploring whether there should be differences in the treatment path between patients with hypertension alone and those with diabetes mellitus and hypertension [12]. Dutch scholars try to find whether the excessive intake of protein in patients with type 1 diabetes (T1DM) is a risk factor for hypertension, but they do not get the correlation between the two [13]. As far as I know, there are few studies on the classification model and determinant factors analysis of hypertension for diabetic patients. Previous literature had confirmed that most diabetes and hypertension could be prevented by reasonable intervention and control [14, 15]. Therefore, it is of great significance to establish a simple and rapid hypertension screening model in patients with T2DM and to improve the detection level of hypertension in auxiliary medical institutions.

The objective of this study is to use easily obtained data to construct a nomogram model to identify T2DM patients with a high likelihood of hypertension. Our approach will be useful in locations with high epidemiological risk and poor socioeconomic status, which do not pay attention to regular blood pressure monitoring, such as Xinjiang, China.

## Materials and methods

### Patient selection

The Xinjiang physical examination is a free physical examination provided by the Chinese government for all Xinjiang people. A total of 643,439 citizens participating in the physical examination of Urumqi in 2018 was recruited, and we had access to information that could identify individual participants during or after data collection. Subjects who met the following inclusion criteria were eligible to participate in the study: (1) age over 20; (2) Type 2 diabetes mellitus (T2DM) patients; (3) participants signed a written informed consent. After a strict data filtration 30,507 were enrolled, finally. The data were randomly divided into development group (n = 21,355) and validation group (n = 9,152). We have used the data from the development set to build the nomograms and used the data from the validation set to verify the model. This study was performed in accordance with the principles outlined in the Declaration of Helsinki and approved by Xinjiang Uygur Autonomous Region CDC ethical committee and the institutional review board.

### Patient characteristics

The Xinjiang physical examination variables include 3 parts: questionnaire, physical examination and laboratory testing. The questionnaire includes information on medical history and lifestyle, such as smoking, drinking, diet and exercise habits. Physical measurement indexes include height, body weight, heart rate, waist circumference and abdominal ultrasound. Abdominal ultrasound can observe the shape and size of the abdominal organs, as well as determine whether these organs have tumors, cysts or stones, including liver, kidney, gallbladder and other organs. Laboratory test indicators include blood glucose and blood biochemistry. In this study, we wanted to establish a simple model that can calculate the possibility of hypertension when it comes to patients with T2DM, only through questionnaire and physical measurement indicators, so this study didn’t include the laboratory test indicators.

### Diagnosis of T2DM

The definition of T2DM in this study was: 2 hours after meal, blood glucose ≥ 11.1mmol/l, fasting blood glucose ≥ 7.0mmol/l, or the main complaint of T2DM and taking hypoglycemic drugs, the final incidence of adult T2DM was 10.5%, excluding gestational diabetes and T1DM. The prevalence was equivalent to the incidence of previous studies [16].

### Diagnosis of hypertension

Blood pressure was measured on both arms of the participants. Hypertension was defined as simultaneous systolic blood pressure ≥ 140 mmHg, simultaneous diastolic blood pressure ≥ 90 mmHg or hypertension with antihypertensive drugs. According to the analysis, the prevalence of hypertension in patients with T2DM included in the study was 43.7%, which was 1.49 times of the prevalence of hypertension in people without T2DM in this study, which was equivalent to the incidence in the previous study [8].

### Risk factors

The potential risk factors used in this study to assess hypertension in patients with T2DM included: age, gender, ethnicity, career, smoking, drinking, exercise, diet habits, obesity, atherosclerotic vascular disease (ASCVD), kidney disease, eye diseases, psychosis, gallbladder_disease, bronchitis and tuberculosis.

Basic information of participants: gender included "male" and "female"; career included "trader or service people", "agriculture workers", "factory workers", "soldier" and "others"; ethnic groups were divided into six categories: "Han", "Uygur", "Kazak", "Hui", "Mongolian" and "other nationalities"; living habits included smoking, drinking, exercise and eating habits. Drinking included drinking/time(g) and drinking frequency: “never”, “occasionally”, “regularly” and “daily”; exercise frequency were divided into four levels:”never”, “occasionally”, “>1 per week” and “daily”; smoking included smoking amount/day (cigarettes) and smoking situation: "non-smoker",”ever-smoker” and “current-smoker”; diet habits were divided into three categories: "meat based", "meat balanced" and "vegetarian based";

In this study, BMI (Body Mass Index) > 28 kg/m^2^ was identified as obesity. The diagnosis of gallbladder disease and kidney diseases were based on the description from the patients and in conjunction with B ultrasonic examination. Gallbladder disease include cholecystitis, cholecystectomy and gallstone, kidney diseases included: diabetic nephropathy, renal failure, acute/chronic nephritis, hydronephrosis, and renal calculus. The other baseline comorbidities considered in this study were determined by health questionnaire survey of morbidities, whether the participant has previously been diagnosed by a doctor. The presence of eye diseases was defined as diagnosis of one or more of the following: retinal hemorrhage, papilledema and cataract; coronary, cerebrovascular or peripheral disease were collectively called ASCVD, participants were asked to answer the question "have you ever had a heart attack, or have you used a stent or taken a bridge?", ASCVD in this study included: myocardial infarction, cerebral infarction, angina pectoris, stroke, cerebral hemorrhage and coronary heart disease. While patients had one or more diseases, the relative variable would be defined as yes, otherwise would be defined as no.

### Statistical analysis

The data of national physical examination are large, and with jumbled variables, existing many missing and abnormal values. So data pre-processing is a very important step, the quality of preprocessing will directly affect the performance of the later prediction models [17]. Firstly, we deleted nearly 200 variables with no meaning to this study. Secondly, we filled in outliers and nulls, classification variables were filled with the most frequent value, and continuous variables were filled with mean value.

While comparing the baseline characteristics between the development group and validation group, differences with a two-sided *p*-value of <0.05 were deemed statistically significant. Categorical variables were presented as the number (percentage). Continuous variables consistent with a normal distribution were presented as mean±standard deviation; otherwise, the median and quartile are used. Chi-square test were used to compare the differences in categorical variables. Independent sample t-tests were used to compare the differences in normal continuous variables, while the Wilcoxon test was used for nonnormal continuous variables.

Lasso regression was used to screen the risk factors. Lasso regression is a kind of shrinkage method in linear regression model. It shrinks the estimated value of uncorrelated variables to close to zero, and then filter out non-zero variables. Lasso combines the advantages of selection process (easy to explain) and expression (robust), which is particularly useful in large data sets requiring efficient and fast algorithms [18]. Analysis steps: Step 1: included 18 effective variables in the data set into the lasso process, and the optimal penalty parameter λ was determined by 10-fold cross validation. Step 2: multivariable logistic regression analysis was used to build a predicting model by incorporating the feature selected in the LASSO regression model. The features were considered as odds ratio (OR) having 95% confidence interval (CI) and as *p*-value. The statistical significance levels were all two-sided. Variables with the *p*-value of 0.05 were included in the model [19, 20]. Finally, the prediction model was evaluated in terms of discrimination and calibration.

The resolution of the prediction model referred to its ability to distinguish people with hypertension from people without hypertension. The area under the curve (AUC) of ROC was used to evaluate whether the prediction results of the model meet the requirements [21]. AUC is usually between 0.5 and 1.0. The closer AUC value is to 1, the stronger the recognition ability of prediction model is [22]. Hosmer-Lemeshow good of fit test was used to evaluate the calibration of the prediction model. If the smaller the chi square value of the statistics is, the larger the corresponding *p* value is, the better the calibration of the prediction model will be. If the test results show statistical significance (*p* < 0.05), it shows that there is a certain difference between the predicted value of the model and the actual observed value, and the model calibration is poor [23].

The open source Python software Version 3.7.2(https://www.python.org) was used for data pre-processing, Pandas library and NumPy library were used for interpolation and processing of outliers, and Matplotlib library was used for data description and outliers judgment; the open source R software Version 3.6.1(http://www.r-project.org) was used for data modeling, CARET-package was used to divide the data into development and validation group randomly, LASSO analysis was performed with glmnet-packages, RMS-package was used to establish a nomogram model, the nomogramEx-package was used to figure the scores of every character in nomogram, the ROC was plotted using ROCR- package and PROC-package, and the calibration curves had been drawn by RMS-package.

## Results

### Patient demographics

A total of 30,507 cases were included in the study, including 21,355 in the development set, 9,152 in the validation set. All data of cases including demographic and disease in the two groups were given in Table 1. The comparison of baseline data showed that there was no significant difference between the development set and the validation set.

**Table 1:** Characteristics of the development set and validation set (n = 30,507).

Characteristics	Development set (n = 21,355)	Validation set (n = 9,152)	*p* value
Age (years)	56.77±17.14	56.95±17.18	0.395
Ethnicity, n (%)			0.079
Han	13871(64.95)	6055(66.16)	
Uygur	3653(17.11)	1543(16.86)	
Kazak	849(3.98)	335(3.66)	
Hui	2388(11.18)	1010(11.04)	
Mongolian	27(0.13)	12(0.13)	
Other nationalities	567(2.66)	197(2.15)	
Gender, n (%)			0.522
Male	10783(50.49)	4658(50.90)	
Female	10572(49.51)	4493(49.10)	
Career, n (%)			0.633
Trader or service people	13138(61.52)	5592(61.11)	
Agriculture workers	6403(29.88)	2791(30.50)	
Factory workers	809(3.79)	341(3.73)	
Soldier	202(0.95)	73(0.80)	
Others	803(3.76)	354(3.87)	
Drinking			
Drinking amount(g)	25(7.50–50)	25(10–50)	0.657
Drinking frequency, n (%)			0.599
Never	17367(81.33)	7401(80.88)	
Occasionally	2239(10.48)	968(10.58)	
Regularly	987(4.62)	455(4.97)	
Daily	762(3.57)	327(3.57)	
Smoking			
Smoking amount (cigarettes)	10(7–10)	10(8–20)	0.060
Smoking situation, n (%)			0.740
Non	18458(86.43)	7936(86.72)	
Ever	573(2.68)	234(2.56)	
Current	2324(10.88)	981(10.72)	
Exercise frequency, n (%)			0.608
Never	10193(47.73)	4342(47.45)	
Occasionally	1542(7.22)	663(7.25)	
>1 per week	858(4.02)	398(4.35)	
Daily	8762(41.03)	3748(40.96)	
Diet habits, n (%)			0.675
Meat based	756(3.54)	310(3.39)	
Meat balanced	20094(94.10)	8614(94.13)	
Vegetarian based	505(2.36)	227(2.48)	
Obesity, n (n)			0.653
No	16711(78.25)	7139(78.01)	
Yes	4644(21.75)	2012(21.99)	
Eye diseases, n (%)			0.457
No	20355(95.32)	8742(95.52)	
Yes	1000(4.68)	410(4.48)	
ASCVD, n (%)			0.624
No	18693(87.53)	7991(87.32)	
Yes	2662(12.47)	1160(12.68)	
Kidney diseases, n (%)			0.092
No	20065(93.96)	8551(93.44)	
Yes	1290(6.04)	600(6.56)	
Psychosis, n (%)			0.380
No	20767(97.25)	8917(97.43)	
Yes	588(2.75)	235(2.57)	
Gallbladder diseases, n (%)			0.408
No	18334(85.85)	7890(86.22)	
Yes	3021(14.15)	1261(13.78)	
Bronchitis, n (%)			0.949
No	20804(97.42)	8913(97.40)	
Yes	551(2.58)	238(2.60)	
Tuberculosis, n (%)			0.555
No	21293(99.71)	9120(99.66)	
Yes	62(0.29)	31(0.34)	

Data are shown as means ± SD, or no. (%). Abbreviations: ASCVD, atherosclerotic vascular disease.

### Characteristics selection

**Fig 1:**
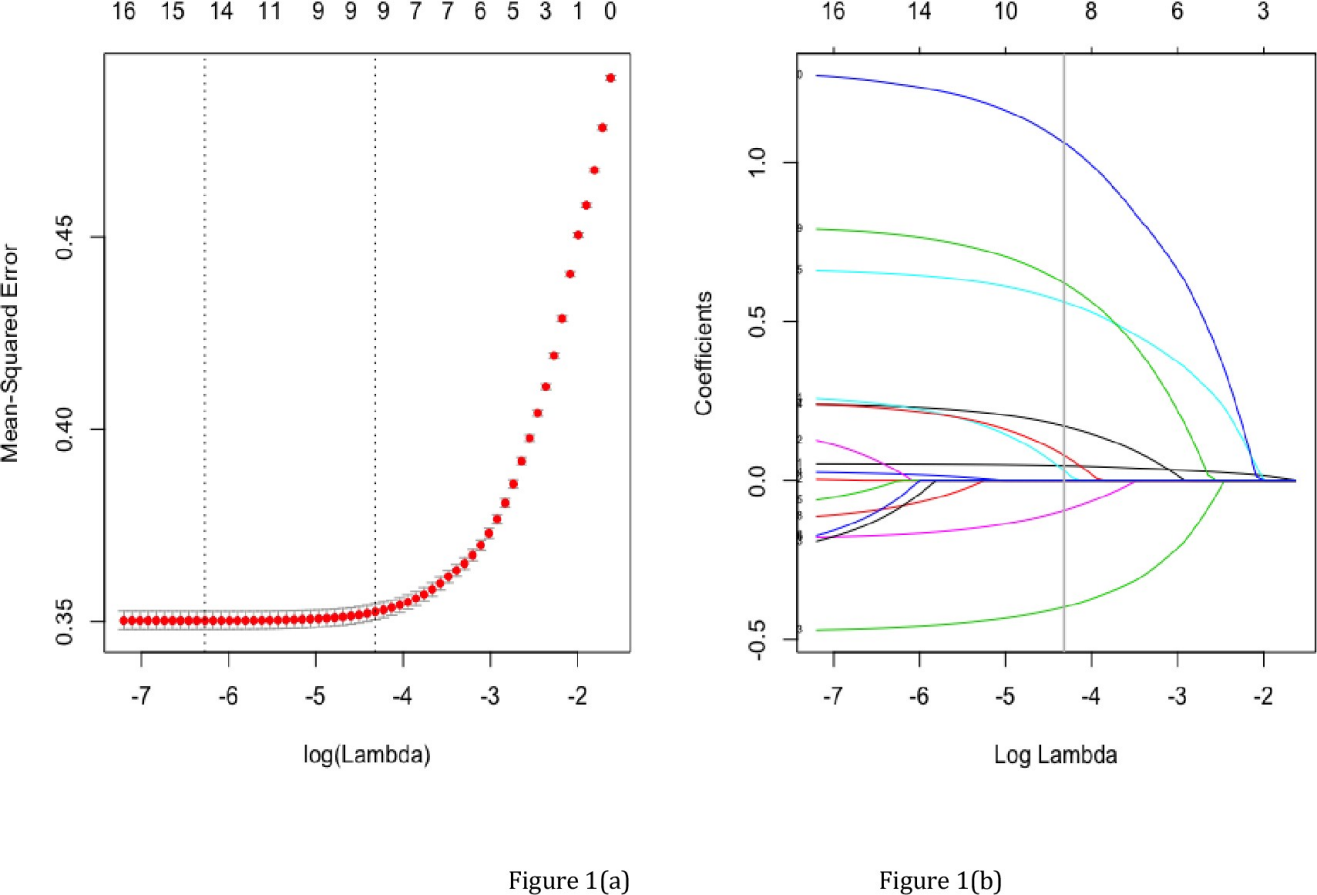
Texture feature selection using the least absolute shrinkage and selection operator (LASSO) binary logistic regression model. (a) Optimization parameters(Lambda) of lasso model were selected by 10 times cross-validation. The Mean-Squared Error was plotted versus log(Lambda). Dotted vertical lines were drawn at the optimal values by using the minimum criteria and the 1 standard error of the minimum criteria (the 1-SE criteria). Minimum criteria refer to the one among all λ values to get the mean value of the minimum target parameter. 1-SE criteria refer to the λ value of the simplest model in a variance range of minimum criteria. (b) LASSO coefficient profiles of the 18 features. A coefficient profile plot was produced against the log Lambda) sequence. Vertical line was drawn at the value selected using 10 times cross-validation, where optimal lambda resulted in 9 features with nonzero coefficients.

Through lasso regression, we got 9 non-zero coefficient characteristics, which showed that we reduced 18 indexes to 9 indexes. As it was shown in Fig 1A and 1B. These features included age, gender, drinking frequency, exercise frequency, smoking situation, obesity, ASCVD, kidney diseases and gallbladder diseases (Table 2).

**Table 2:** Multivariate logistic regression analysis for risk factors associated with hypertension in the training cohort (N = 21,355).

Variable	β	Odds Ratio	95% CI	Z value	P value
Age	0.054	1.055	(1.053–1.058)	44.630	<0.001
Gender					
Male	Ref	1	Ref	—	—
Female	-0.479	0.619	(0.576–0.665)	-13.084	<0.001
Drinking frequency					
Never	Ref	1	Ref	—	—
Occasionally	0.256	1.292	(1.147–1.455)	4.209	<0.001
Regularly	1.884	6.578	(5.425–8.030)	18.846	<0.001
Daily	1.937	6.937	(5.538–8.779)	16.499	<0.001
Exercise frequency					
Never	Ref	1	Ref	—	—
Occasionally	-0.514	0.598	(0.521–0.686)	-7.314	<0.001
>1 per week	-0.508	0.602	(0.507–0.713)	-5.835	<0.001
Daily	-0.530	0.588	(0.547–0.633)	-14.146	<0.001
Smoking situation					
Non	Ref	1	Ref	—	—
Ever	-0.080	0.923	(0.755–1.130)	-0.777	0.437
Current	0.699	2.011	(1.782–2.270)	11.322	<0.001
Obesity					
No	Ref	1	Ref	—	—
Yes	0.802	2.229	(2.064–2.408)	20.388	<0.001
ASCVD					
No	Ref	1	Ref	—	—
Yes	1.342	3.826	(3.412–4.296)	22.833	<0.001
Kidney diseases					
No	Ref	1	Ref	—	—
Yes	0.280	1.323	(1.155–1.515)	4.044	<0.001
Tuberculosis					
No	Ref	1	Ref	—	—
Yes	0.237	1.268	(1.156–1.390)	5.033	<0.001

β is the regression coefficient, CI = Confidence Interval. Abbreviations: ASCVD, atherosclerotic vascular disease; OR, odds ratio; CI, confidence interval.

### Independent prognostic factors in the developing set

The 9 variables obtained by lasso regression were included in logistic multiple regression model, and the regression results were shown in Table 2. Through model analysis, we knew that: age(OR 1.06), gender(female, OR 0.62), drinking frequency(occasionally: OR 1.29, regularly: OR 6.58, daily: OR 6.94), exercise frequency(occasionally: OR 0.60, >1 per week: OR 0.60, daily: OR 0.59), smoking situation(ever: OR 0.92, current: OR 2.01), obesity(yes: OR 2.23), ASCVD(yes: OR 3.83), kidney diseases(yes: OR 1.32), tuberculosis(yes: OR 1.27) were independent determinant factors of hypertension (Table 2). In addition, there was no evidence of multicol-linearity among the covariates included in the model. Maximum VIF(variance inflation factor) was 1.245, and lowest eigen value was 1.003.

### Nomogram of hypertension

Based on the logistic multiple regression, OR of categorical variables kidney diseases and tuberculosis were closed to 1, indicating that these variables had less influences, so we didn’t take theses in the model.

**Fig 2:**
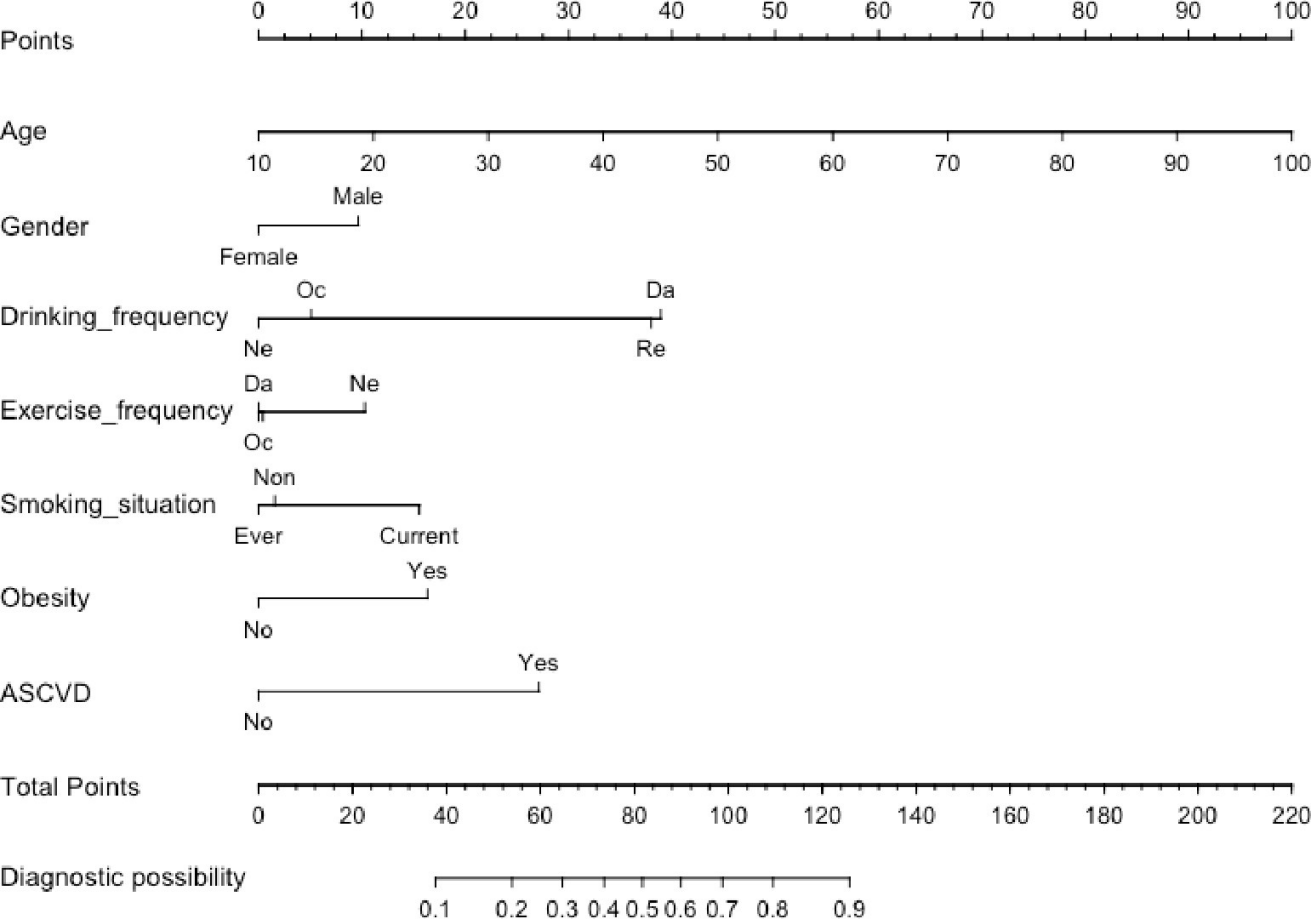
Nomogram to predict the presence of hypertension in T2DM patients. Abbreviations: ASCVD, atherosclerotic vascular disease. To use the nomogram, an individual participants value is located on each variable axis, and a line is drawn upward to determine the number of points received for each variable value. The sum of these numbers is located on the Total Points axis to determine the risk of hypertension.

Finally, we got Urumqi Hypertension Nomogram Model consisting of 7 factors (Fig 2). The longer the length of line, the greater the impact of risk factors on the effectiveness rate of hypertension. Each sub-type in these variables is assigned a score. The cumulative sum of each “point” is the “total points”. The corresponding “diagnostic possibility” of “total point” is the predicted probability of hypertension suggested by our designed nomogram.

Take an example of nomogram usage: a sample was randomly selected from the subjects. A 40 years old man with diabetes, regular drinking, exercise occasionally, no smoking, with ASCVD but without obesity history, and we can calculate that his total score was 109.66, and the probability of hypertension was close to 80%.

### Validation of the nomogram

**Fig 3:**
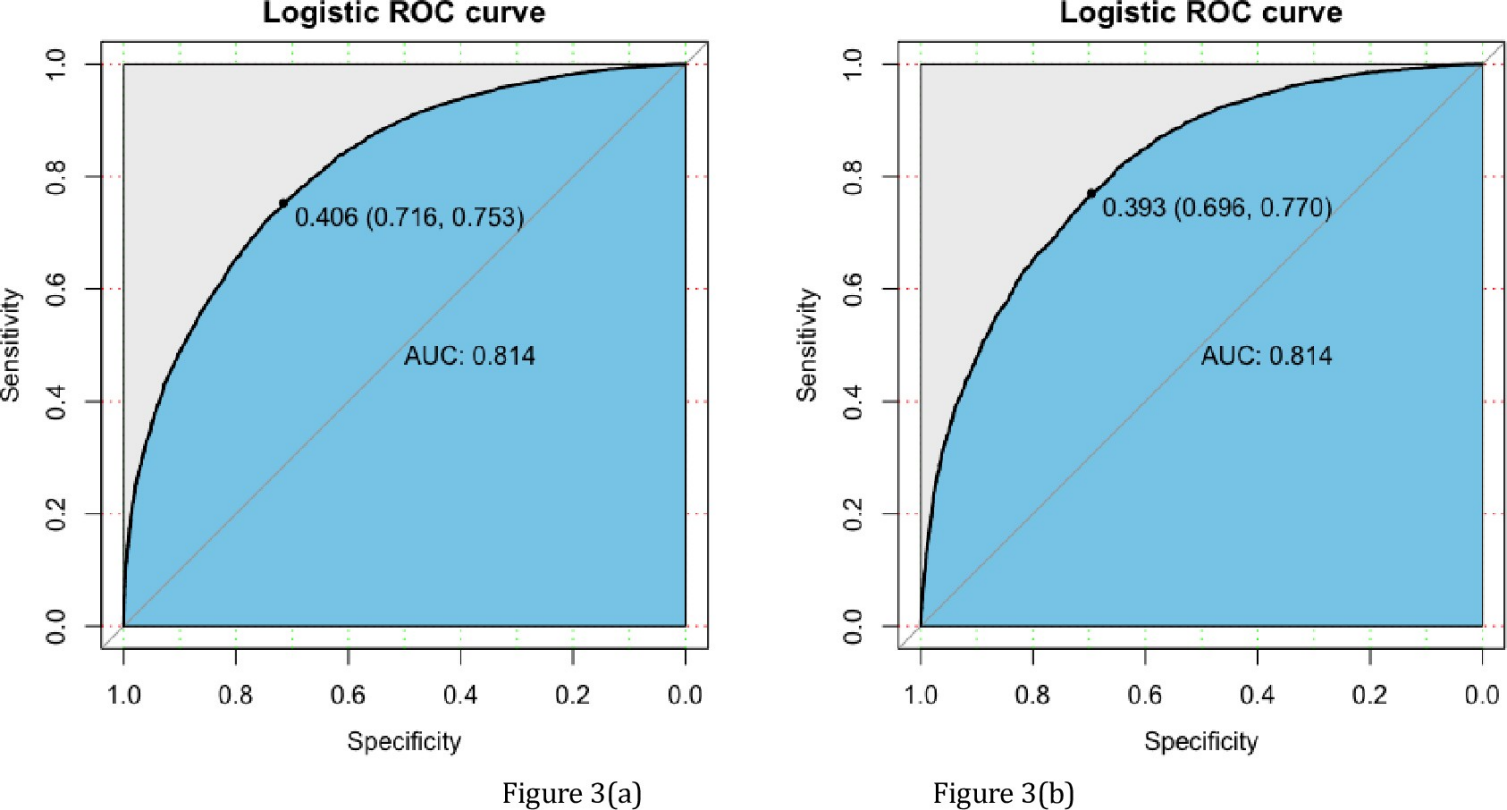
ROC curves for validating the discrimination power of the nomogram. (a) Development group. (b) Validation group. (AUC = 0.814).

**Fig 4:**
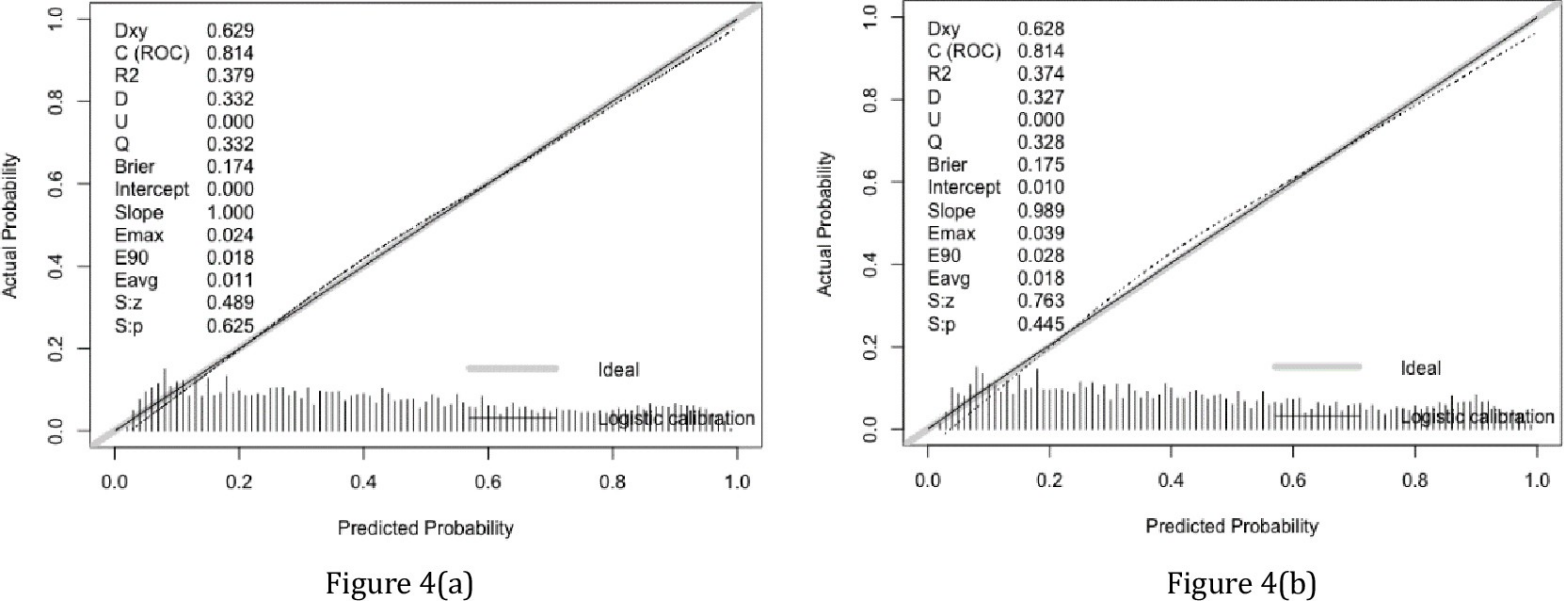
Calibration curves for the prediction and training group models (*p* = 0.625 vs 0.445). The diagonal solid line represents a perfect prediction by an ideal model. The dotted line represents the performance of the nomogram, of which a closer fit to the diagonal dotted line represents a better prediction.

The validation of the model was based on discrimination and calibration. Plot prediction accuracy ROC, and calculate AUC value of development and validation group, respectively. And the AUC value of development group and validation group were both 0.814 (Fig 3A and 3B), indicating that nomogram prediction model had good discrimination ability. The calibration of the prediction model was evaluated by Hosmer-Lemeshow good of fit test, and the calibration curve (Fig 4A and 4B) was obtained. When *p*>0.05, the calibration ability of the model is good. The calibration curve of the development group was *p* = 0.625, and the calibration curve of the validation group was *p* = 0.445, all of which were greater than 0.05, indicating that the model had good calibration ability.

## Discussion

Diabetes and high blood pressure have become the main chronic diseases endangering the health of Chinese adults [23–25]. Hypertension and diabetes are the main manifestations of metabolic syndrome. The results of this study show that the prevalence of hypertension in patients with T2DM in Urumqi, Xinjiang, China is 43.7%, which was 1.49 times higher than that in patients without diabetes. We established a nomogram to predict the possibility of hypertension, with the feature of user-friendly digital interfaces, increased accuracy, and more easily understood prognoses, which can be widely used in prognostic devices in oncology and medicine and this would be the first to fit the nomogram model for the study of determinant factors of hypertension with T2DM.

Studies have shown that diabetes can accelerate the hardening of the aorta, reduce its own compliance, reduce its elastic expansion ability, and increase the systolic pressure; diabetic peripheral nerve damage can cause microvascular contraction dysfunction and change the diastolic pressure [26, 27]. This may be because hypertension and diabetes are both rooted in the same soil as insulin resistance [28, 29]. In the early stage of insulin resistance, hyperinsulinemia can increase sympathetic nerve activity by promoting the reabsorption of sodium in renal tubules, accelerate heart rate, increase vascular resistance, promote the proliferation of smooth muscle of arterioles, lead to lumen stenosis, increase intracellular calcium concentration, and be more sensitive to pressor substances, so as to increase blood pressure. T2DM and hypertension are viewed as homologous diseases. Both diseases share the same etiology, mutual influence and harm. In recent years, epidemiological studies have clarified the role and pathogenesis of microcirculation disorders in diabetic patients [30], which is more closely related to multiple organs and body systems, including the heart, brain, blood vessels, kidneys, eyes and nerves [31, 32].

Besides, Rahul Aggarwal reported that the combination of T2DM and hypertension is more likely to be associated with person who is older, male, higher BMI and less physical activity [33], a reasonable diet, exercise, weight control, and central obesity control are useful in preventing hypertension [34–37]. Previous studies have also provided us with evidence of aggravation of organ damage caused by alcohol in diabetic patients [38]. Different from that in healthy people, diabetic patients should abstain from alcohol. Besides, we found that smoking is the risk factor of hypertension, and the harm of nicotine to human endocrine system and vascular system has already been proved [39]. Therefore, we can control the occurrence and development of hypertension by quit drinking and smoking, strengthening exercise, and losing weight. Our study found that the possibility of developing hypertension is related to the degree of smoking, drinking and exercising, suggesting that although we now smoke, quitting smoking is beneficial to disease control. The higher the frequency of drinking, the more likely we are to develop the disease, suggesting that although we may not be able to abstain completely, reducing the frequency of drinking may also be beneficial in controlling the disease.

However, some known risk factors for hypertension, such as unhealthy diet [40], were not associated with the incidence of hypertension in this study. The reason for this result may be that compared with the general population, diabetic patients may focus on healthy living habits, which may due to the ability of diabetic patients to get more medical attention and more information about health improvement measures, such as diabetics tended to control blood sugar by reducing the intake of high-fat and high-oil foods [41].

Diabetes and hypertension are considered to be the main risk factors for cardiovascular disease and stroke [42, 43], and the mortality rate of cardiovascular disease in T2DM and hypertension patients is higher than that in people with two diseases alone [44]. In view of the increasingly severe trend of diabetes and hypertension in recent years, we developed a nomogram to identity of hypertension in patients with T2DM. This nomogram is very intuitive, so diabetic patients can easily calculate their probability of hypertension without the help of nursing staff. This research has several advantages. First, as far as we know, this is the first model to develop and evaluate the possibility of hypertension in patients with T2DM. Second, we used a national database to identify a large and representative sample of national health examination. Third, The variables we used were all from the questionnaire, and there was no need to measure any indicators (such as blood pressure, blood routine, height, weight, etc.). The variables were very easy to obtain, and this method was especially effective in areas with poor medical treatment and lacking attention to blood pressure measurement. Although it is very easy to measure blood pressure, there are still some regions, where regular blood pressure measurement is not completely universal. Therefore, our study can be used as an effective auxiliary diagnostic tool for hypertension. Fourth, it includes four adjustable variables: smoking, drinking, exercise habits and obesity, which can encourage people to prevent diseases through a healthy lifestyle, as studies have shown that healthy lifestyle is an economical and effective means to prevent chronic diseases [45, 46].

We did not include the family history information in our model, and many people suffer from undiagnosed diabetes or hypertension [47], given the inadequacy of China's health system in the last century. As a result, many participants were uncertain about their previous generation's medical history, and family history parameters did not improve risk prediction, so we did not collect data on family history.

This study also has some limitations. First of all, our study is a cross-sectional study, which fails to determine the cause and effect of the disease and couldn’t know the interaction between diabetes and hypertension. Second, we are unable to collect data on other potential risk factors, such as intake of sodium and potassium in the diet. Third, the data used in this study is the physical examination data of Urumqi, China, which may limit the extrapolation of the results. It is generally believed that there are some differences in the pathophysiology of diseases between Asians and Caucasians, and there are similar differences between Asian countries. Fourth, previous studies have confirmed that education are also important determinants of hypertension. However, our physical examination data failed to obtain the education of participants. Finally, this cross-sectional study can not predict the disease. However, the model can obtain the probability that an individual has a disease, and the risk factors can remind people to improve their living habits and control the occurrence and development of the disease.

## Conclusion

In view of the increasingly serious harm of diabetes mellitus combined with hypertension to human beings, based on a large-scale physical examination population, this study used questionnaire variables to establish a screening model, and obtained the risk factors of the disease, and high accuracy of the model. Based on these results, we developed a nomogram, which could easily and quickly calculate the possibility of an individual suffering from a disease, this method is useful especially in those areas with poor medical condition, which can also encourage people to live a healthy life to prevent the occurrence of disease. However, further studies are needed to develop models that more precisely predict various comorbidities (including hypertension) and support preventive guidelines and interventions for patients who have survived T2DM.

## References

[1] Chen Z. Launch of the health-care reform plan in China. Lancet. 2009;373(9672):1322–4. 10.1016/S0140-6736(09)60753-4 19376436

[2] Rahelić D. 7TH EDITION OF IDF DIABETES ATLAS—CALL FOR IMMEDIATE ACTION. Lijec Vjesn. 2016;138(1–2):57–8. https://www.ncbi.nlm.nih.gov/pubmed/27290816. 27290816

[3] Wang Z, Chen Z, Zhang L, Wang X, Hao G, Zhang Z, et al Status of Hypertension in China: Results From the China Hypertension Survey, 2012–2015. Circulation. 2018;137(22):2344–56. 10.1161/CIRCULATIONAHA.117.032380 29449338

[4] Lu J, Lu Y, Wang X, Li X, Linderman GC, Wu C, et al Prevalence, awareness, treatment, and control of hypertension in China: data from 1·7 million adults in a population-based screening study (China PEACE Million Persons Project). Lancet. 2017;390(10112):2549–58. 10.1016/S0140-6736(17)32478-9 29102084

[5] Oktay AA, Akturk HK, Jahangir E. Diabetes mellitus and hypertension: a dual threat. Curr Opin Cardiol. 2016;31(4):402–9. 10.1097/HCO.0000000000000297 27070651

[6] Han C, Rice MW, Cai D. Neuroinflammatory and autonomic mechanisms in diabetes and hypertension. Am J Physiol Endocrinol Metab. 2016;311(1):E32–E41. 10.1152/ajpendo.00012.2016 27166279 PMC4967151

[7] Liu J, Zhao D, Liu J, Qi Y, Sun J, Wang W. Prevalence of diabetes mellitus in outpatients with essential hypertension in China: a cross-sectional study. BMJ Open. 2013;3(11):e003798–e. 10.1136/bmjopen-2013-003798 24259390 PMC3840347

[8] Ji L, Hu D, Pan C, Weng J, Huo Y, Ma C, et al Primacy of the 3B approach to control risk factors for cardiovascular disease in type 2 diabetes patients. Am J Med. 2013;126(10):925.e11–.e9.25E22. 10.1016/j.amjmed.2013.02.035 23810406

[9] Cryer MJ, Horani T, DiPette DJ. Diabetes and Hypertension: A Comparative Review of Current Guidelines. J Clin Hypertens (Greenwich). 2016;18(2):95–100. 10.1111/jch.12638 26234374 PMC8031521

[10] Tranquilli AL. Hypertension during pregnancy is associated with increased risk of later cardiovascular disease, kidney disease and diabetes. Evid Based Nurs. 2014;17(2):36–7. 10.1136/eb-2013-101322 23828761

[11] Yano Y, Reis JP, Colangelo LA, Shimbo D, Viera AJ, Allen NB, et al Association of Blood Pressure Classification in Young Adults Using the 2017 American College of Cardiology/American Heart Association Blood Pressure Guideline With Cardiovascular Events Later in Life. JAMA. 2018;320(17):1774–82. 10.1001/jama.2018.13551 30398601 PMC6248102

[12] Thomopoulos C, Parati G, Zanchetti A. Effects of blood-pressure-lowering treatment on outcome incidence in hypertension: 10—Should blood pressure management differ in hypertensive patients with and without diabetes mellitus? Overview and meta-analyses of randomized trials. J Hypertens. 2017;35(5):922–44. 10.1097/HJH.0000000000001276 28141660

[13] Altorf-van der Kuil W, Engberink MF, Ijpma I, Verberne LDM, Toeller M, Chaturvedi N, et al Protein intake in relation to risk of hypertension and microalbuminuria in patients with type 1 diabetes: the EURODIAB Prospective Complications Study. J Hypertens. 2013;31(6):1151–9. 10.1097/HJH.0b013e328360418e 23524911

[14] Carey RM, Muntner P, Bosworth HB, Whelton PK. Prevention and Control of Hypertension: JACC Health Promotion Series. J Am Coll Cardiol. 2018;72(11):1278–93. 10.1016/j.jacc.2018.07.008 30190007 PMC6481176

[15] Tuomilehto J, Lindström J, Eriksson JG, Valle TT, Hämäläinen H, Ilanne-Parikka P, et al Prevention of type 2 diabetes mellitus by changes in lifestyle among subjects with impaired glucose tolerance. N Engl J Med. 2001;344(18):1343–50. 10.1056/NEJM200105033441801 11333990

[16] Gao Y, Xie X, Wang S-X, Li H, Tang H-Z, Zhang J, et al Effects of sedentary occupations on type 2 diabetes and hypertension in different ethnic groups in North West China. Diab Vasc Dis Res. 2017;14(4):372–5. 10.1177/1479164117696050 28622744

[17] Ngiam KY, Khor IW. Big data and machine learning algorithms for health-care delivery. Lancet Oncol. 2019;20(5):e262–e73. 10.1016/S1470-2045(19)30149-4 31044724

[18] Mueller-Using S, Feldt T, Sarfo FS, Eberhardt KA. Factors associated with performing tuberculosis screening of HIV-positive patients in Ghana: LASSO-based predictor selection in a large public health data set. BMC Public Health. 2016;16:563–. 10.1186/s12889-016-3239-y 27412114 PMC4944423

[19] Iasonos A, Schrag D, Raj GV, Panageas KS. How to build and interpret a nomogram for cancer prognosis. J Clin Oncol. 2008;26(8):1364–70. 10.1200/JCO.2007.12.9791 18323559

[20] Silva TB, Oliveira CZ, Faria EF, Mauad EC, Syrjänen KJ, Carvalho AL. Development and validation of a nomogram to estimate the risk of prostate cancer in Brazil. Anticancer Res. 2015;35(5):2881–6. https://www.ncbi.nlm.nih.gov/pubmed/25964571. 25964571

[21] Harrell FE Jr, Califf RM, Pryor DB, Lee KL, Rosati RA. Evaluating the yield of medical tests. JAMA. 1982;247(18):2543–6. https://www.ncbi.nlm.nih.gov/pubmed/7069920. 7069920

[22] Niu XK, He WF, Zhang Y, Das SK, Li J, Xiong Y, et al Developing a new PI-RADS v2-based nomogram for forecasting high-grade prostate cancer. Clin Radiol. 2017;72(6):458–64. 10.1016/j.crad.2016.12.005 28069159

[23] Kramer AA, Zimmerman JE. Assessing the calibration of mortality benchmarks in critical care: The Hosmer-Lemeshow test revisited. Crit Care Med. 2007;35(9):2052–6. 10.1097/01.CCM.0000275267.64078.B0 17568333

[24] Nilsson PM, Cederholm J. Diabetes, hypertension, and outcome studies: overview 2010. Diabetes Care. 2011;34 Suppl 2(Suppl 2):S109–13. 10.2337/dc11-s204 21525440 PMC3632146

[25] Torp-Pedersen C, Jeppesen J. Diabetes and hypertension and atherosclerotic cardiovascular disease: related or separate entities often found together. Hypertension. 2011;57(5):887–8. 10.1161/HYPERTENSIONAHA.110.168583 21403088

[26] Libianto R, Batu D, MacIsaac RJ, Cooper ME, Ekinci EI. Pathophysiological Links Between Diabetes and Blood Pressure. Can J Cardiol. 2018;34(5):585–94. 10.1016/j.cjca.2018.01.010 29731021

[27] Verdecchia P, Angeli F. Natural history of hypertension subtypes. Circulation. 2005;111(9):1094–6. 10.1161/01.CIR.0000158690.78503.5F 15753224

[28] Saladini F, Dorigatti F, Santonastaso M, Mos L, Ragazzo F, Bortolazzi A, et al Natural history of hypertension subtypes in young and middle-age adults. Am J Hypertens. 2009;22(5):531–7. 10.1038/ajh.2009.21 19229194

[29] Reaven GM. Relationships among insulin resistance, type 2 diabetes, essential hypertension, and cardiovascular disease: similarities and differences. J Clin Hypertens (Greenwich). 2011;13(4):238–43. 10.1111/j.1751-7176.2011.00439.x 21466618 PMC8673405

[30] Strain WD, Paldánius PM. Diabetes, cardiovascular disease and the microcirculation. Cardiovasc Diabetol. 2018;17(1):57–. 10.1186/s12933-018-0703-2 29669543 PMC5905152

[31] Trudeau L, Gilbert J. Diabetes and Hypertension: The Low and High Points. Can J Diabetes. 2018;42(2):113–4. 10.1016/j.jcjd.2018.01.007 29602403

[32] Nagy K, Sipos E, El Hadj Othmane T. Heart rate variability is significantly reduced in non-diabetic patients with hypertension. Orv Hetil. 2014;155(22):865–70. 10.1556/OH.2014.29886 24860051

[33] Aggarwal R, Steinkamp J, Chiu N, Petrie B, Mirzan H. Intensive Blood Pressure Targets for Diabetic and Other High-Risk Populations: A Pooled Individual Patient Data Analysis. Hypertension. 2018;71(5):833–9. 10.1161/HYPERTENSIONAHA.117.10713 29531175

[34] Siscovick DS, Barringer TA, Fretts AM, Wu JHY, Lichtenstein AH, Costello RB, et al Omega-3 Polyunsaturated Fatty Acid (Fish Oil) Supplementation and the Prevention of Clinical Cardiovascular Disease: A Science Advisory From the American Heart Association. Circulation. 2017;135(15):e867–e84. 10.1161/CIR.0000000000000482 28289069 PMC6903779

[35] Yamagishi K, Hosoda T, Sairenchi T, Mori K, Tomita H, Nishimura A, et al Body mass index and subsequent risk of hypertension, diabetes and hypercholesterolemia in a population-based sample of Japanese. Nihon Koshu Eisei Zasshi. 2003;50(11):1050–7. https://www.ncbi.nlm.nih.gov/pubmed/14699858. 14699858

[36] Yeh CJ, Pan WH, Jong YS, Kuo YY, Lo CH. Incidence and predictors of isolated systolic hypertension and isolated diastolic hypertension in Taiwan. J Formos Med Assoc. 2001;100(10):668–75. https://www.ncbi.nlm.nih.gov/pubmed/11760372. 11760372

[37] Börjesson M, Onerup A, Lundqvist S, Dahlöf B. Physical activity and exercise lower blood pressure in individuals with hypertension: narrative review of 27 RCTs. Br J Sports Med. 2016;50(6):356–61. 10.1136/bjsports-2015-095786 26787705

[38] Munukutla S, Pan G, Deshpande M, Thandavarayan RA, Krishnamurthy P, Palaniyandi SS. Alcohol Toxicity in Diabetes and Its Complications: A Double Trouble? Alcohol Clin Exp Res. 2016;40(4):686–97. 10.1111/acer.13008 27013182

[39] Basu S, Sussman JB, Rigdon J, Steimle L, Denton BT, Hayward RA. Benefit and harm of intensive blood pressure treatment: Derivation and validation of risk models using data from the SPRINT and ACCORD trials. PLoS Med. 2017;14(10):e1002410 10.1371/journal.pmed.1002410 29040268 PMC5644999

[40] Schwingshackl L, Schwedhelm C, Hoffmann G, Knüppel S, Iqbal K, Andriolo V, et al Food Groups and Risk of Hypertension: A Systematic Review and Dose-Response Meta-Analysis of Prospective Studies. Adv Nutr. 2017;8(6):793–803. 10.3945/an.117.017178 29141965 PMC5683007

[41] Chester B, Babu JR, Greene MW, Geetha T. The effects of popular diets on type 2 diabetes management. Diabetes Metab Res Rev. 2019;35(8):e3188 10.1002/dmrr.3188 31121637

[42] Schernthaner G. Hypertension, insulin resistance and diabetes mellitus: pathophysiological interactions and therapeutic consequences. Wien Klin Wochenschr. 1990;102(24):707–12. https://www.ncbi.nlm.nih.gov/pubmed/1980767. 1980767

[43] Lewington S, Clarke R, Qizilbash N, Peto R, Collins R, Prospective Studies C. Age-specific relevance of usual blood pressure to vascular mortality: a meta-analysis of individual data for one million adults in 61 prospective studies. Lancet. 2002;360(9349):1903–13. 10.1016/s0140-6736(02)11911-8 12493255

[44] Lim N-K, Son K-H, Lee K-S, Park H-Y, Cho M-C. Predicting the risk of incident hypertension in a Korean middle-aged population: Korean genome and epidemiology study. J Clin Hypertens (Greenwich). 2013;15(5):344–9. 10.1111/jch.12080 23614850 PMC8033843

[45] Diehl K, Gansefort D, Herr RM, Görig T, Bock C, Mayer M, et al Physician Gender and Lifestyle Counselling to Prevent Cardiovascular Disease: A Nationwide Representative Study. J Public Health Res. 2015;4(2):534 10.4081/jphr.2015.534 26425495 PMC4568424

[46] Lelong H, Blacher J, Baudry J, Adriouch S, Galan P, Fezeu L, et al Combination of Healthy Lifestyle Factors on the Risk of Hypertension in a Large Cohort of French Adults. Nutrients. 2019;11(7). 10.3390/nu11071687 31340445 PMC6683281

[47] Wang K, Gong M, Xie S, Zhang M, Zheng H, Zhao X, et al Nomogram prediction for the 3-year risk of type 2 diabetes in healthy mainland China residents. Epma j. 2019;10(3):227–37. 10.1007/s13167-019-00181-2 31462940 PMC6695459

